# Evolutionary game analysis of opportunistic behavior of Sponge City PPP projects: a perceived value perspective

**DOI:** 10.1038/s41598-022-12830-0

**Published:** 2022-05-25

**Authors:** Hui Zhao, Xin Liu, Yiting Wang

**Affiliations:** School of Management Engineering, Qingdao University of Technology, Qingdao, 266520 China

**Keywords:** Engineering, Sustainability

## Abstract

Sponge City Public Private Partnership (PPP) project is a significant step to promote the construction of resilient city and sustainable development. Private companies take advantage of information asymmetry and regulatory loopholes to take opportunistic behavior, which affects the project delivery quality and public interests. In order to reveal the decision-making mechanism of the main stakeholders, this paper constructs an evolutionary game model of private companies, citizens and the government from a fresh perspective of perceived value. First, the traditional payoff matrix is modified by combining Prospect Theory and Mental Accounting. Next, this paper analyzes the strategic evolution law and stability conditions of game players by replicated dynamic equation. Finally, Nanganqu project is used for empirical simulation to verify the effectiveness of this model. Results indicate that, (1) due to the complexity of the project and the bounded rationality of the participants, there is no evolutionary stable strategy in this game system. (2) The behavioral decision of participants is affected by perceived incomes and perceived costs. (3) Government punishment and reputation loss can effectively curb the opportunistic behavior. All above studies are expected to improve the management of Sponge City PPP projects, providing theoretical guidance for the government to make scientific decisions.

## Introduction

**Figure 1:**
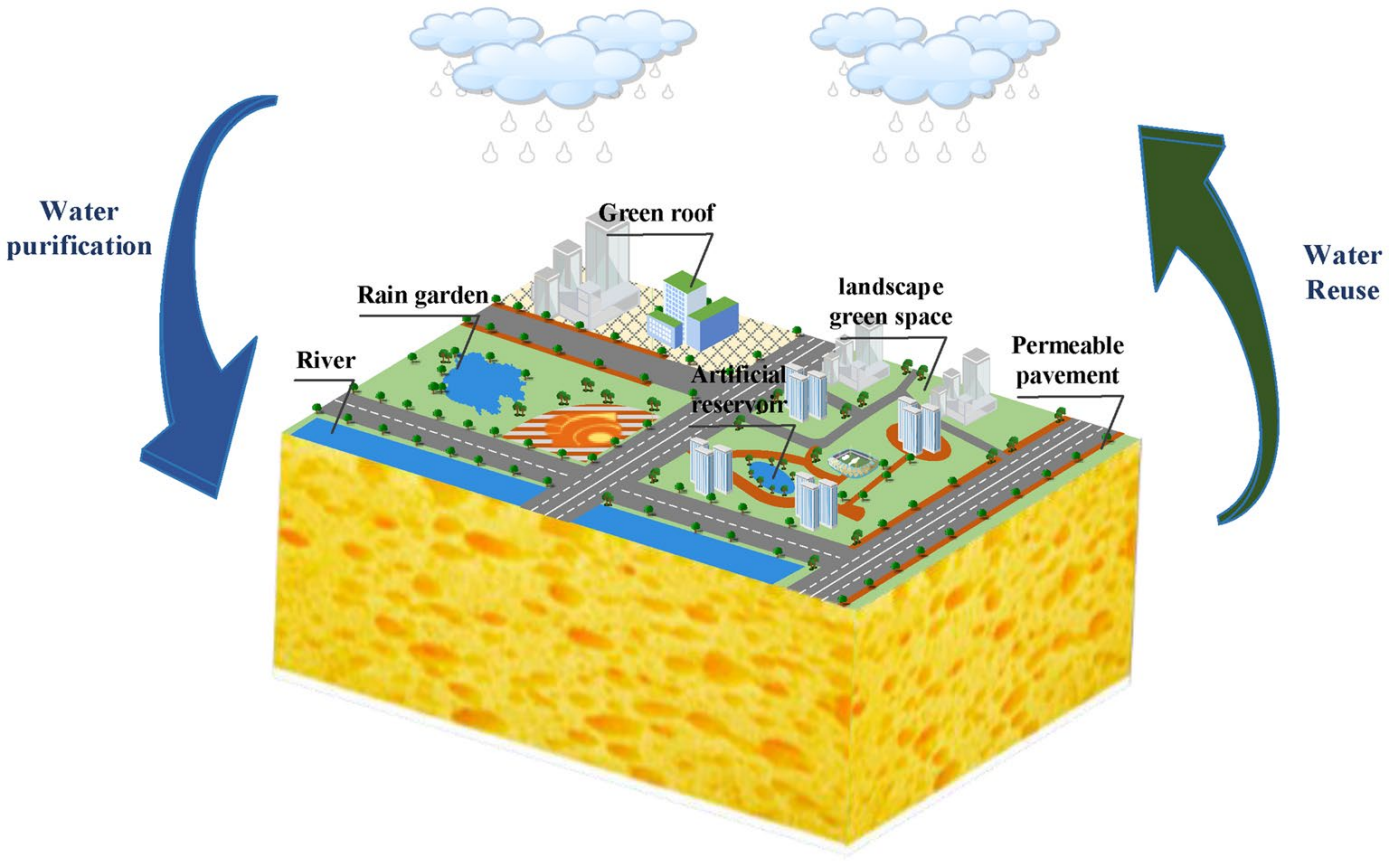
Sketch of sponge city.

**Figure 2:**
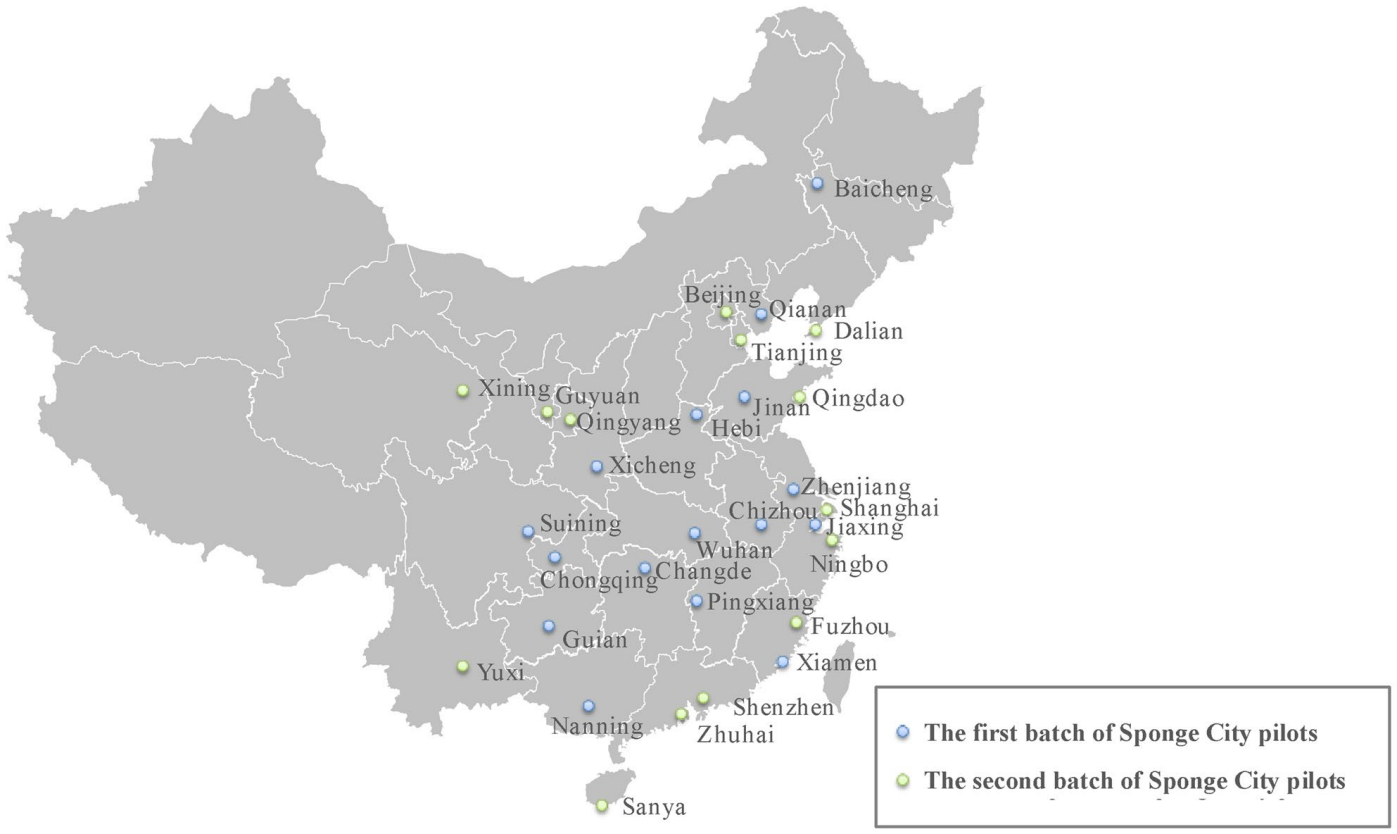
Statistics of Sponge City distribution.

**Figure 3:**
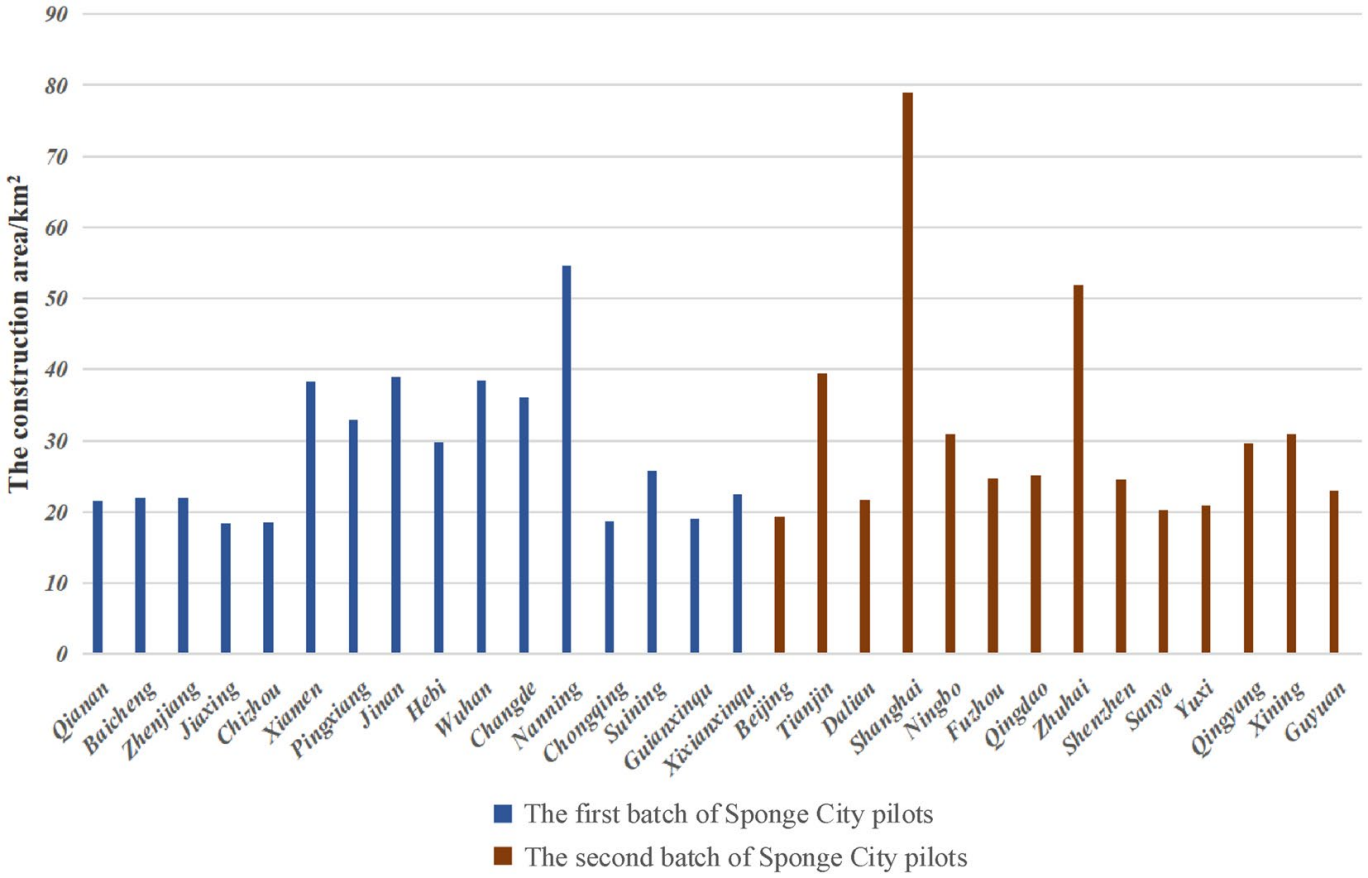
The construction area of Sponge City projects in China.

With the rapid development of society and the continuous improvement of urbanization rate, the ecological and environmental problems are becoming increasingly prominent, especially water problems (such as water environment damage, urban waterlogging, deterioration of water quality, etc.), which have seriously restricted the sustainable development of urban ecology^1,2^ and have received increasingly worldwide concern^3,4^. Under the background of global climate change, extreme events such as extreme high temperature, extreme precipitation and floods will occur more frequently in cities in the future, which puts forward higher requirements for the resilience of urban water system^5^. In China, in order to solve urban water problems, Chinese President Xi Jinping emphasized the construction of Sponge City (SC) Program (Fig. 1) with “natural accumulation, natural infiltration and natural purification” in the Central Urbanization Work Conference^6,7^. SC aims to effectively realize the benign water cycle within the city by “absorbing, storing, infiltrating and purifying” rainwater resources. It is an important benefit project related to water ecology, water environment and water security, and plays an important role in enhancing the water resilience of the urban system^8^. During 2015 and 2016, two batches of 30 cities have been selected as pilots for implementing SC initiative^9^, as shown in Fig. 2. According to statistics from the Ministry of Finance, the investment in SC construction is about 29 million US dollars per square kilometer. With the advancement of SC projects, it is difficult to meet the construction demand solely relying on government financial investment^10^. Specific implementation details of the construction area are given in Fig. 3^11^. In order to solve the problem of huge capital demand, the Public Private Partnership (PPP) mode, in which the government and private companies jointly participate in investment, construction, operation and management, is highly valued by the Chinese government and is also an important part of the construction of SC^12^. The construction of SC projects through PPP mode will help to improve the project’s own financing capacity and solve the problem of insufficient funds, and can give full play to the extensive management and construction experience of private companies, which helps to improve the supply quality of SC projects^13^. In general, SC PPP projects are in line with the era background of sustainable development of ecological civilization, and are an important direction of urban renewal construction invested by the Chinese government.

Although PPP mode provides an effective way to promote the construction of SC, there are potential opportunistic risks hidden in its actual operation. Compared with other projects, SC PPP projects have the characteristics of multiple participating departments, strong public welfare, and long construction period, which leads to the complexity and difficulty in controlling the project participants and is the main sticking point in promoting the further development of SC projects^10^. As the main stakeholders of SC projects, the expected results between the government and private companies are different^14^. The government’s expectation is to maximize public benefits by providing goods or services that meet public needs, while private companies expect to maximize its own benefits^15^. This difference creates hidden dangers of the occurrence of opportunistic behavior^16^. In addition, although the government has set strict standards such as technical standards, performance appraisals, etc., to restrict the construction process of SC projects. However, due to the incompleteness of contracts and the information asymmetry between the main participants, it has created conditions for the occurrence of opportunistic behavior^17^.Thus, the issue towards how to effectively control the opportunistic behavior in the operation of SC PPP projects is an urgent problem perplexes governments. After years of engineering practice, some problems have gradually emerged in the construction SC programs. For example, “severe urban flooding in 19 SC in China” (including megacities of Shanghai, and Beijing) reported by Xinhua^18^ has aroused widespread concern^19^. In July 2021, Zhengzhou, a city investing 50 billion RMB to build SC projects, failed to cope with a rainstorm that resulted in 25 deaths, raising public doubts about the effectiveness of construction of SC projects^20^. The main reason is that the government focuses on system technology rather than construction management, which is not conducive to the regulation of opportunistic behavior^12^. Opportunistic behavior affects the delivery quality of SC PPP projects and also causes the loss of state funds, resulting in damage to the rights and interests of the public^15^. Therefore, the governance of opportunistic behavior is of great significance in the sound operation of SC PPP projects and urban resilience.

The construction of SC is a renovation and upgrading of the urban water management system, a sustainable engineering project that needs to stand the test of time^9^. Mills^21^ presented that the supervision mechanism can significantly affect the opportunistic behavior of private companies. Jiang et al.^22^ held the same view and pointed out that the public attributes of SC PPP projects make it necessary for the government to assume regulatory responsibility. Meanwhile, the public, as the beneficiary of the results of SC projects, also has the right to exercise public supervision over the project operation^23^. Wang et al.^24^ agreed and acknowledged that public attitudes are critical to the follow-up of the projects. Therefore, it can be seen that private companies, citizens and the government play an important role in the operation of SC projects. At present, China is still in the preliminary exploration stage of SC construction, and lacks effective reference cases and normative guidance for localization construction^20^. In order to ensure the construction quality of SC projects and realize the target of sustainable relationship between human and natural ecosystems, there is an urgent need to conduct in-depth analysis of the evolution mechanism of opportunistic behavior.

Previous studies related to the opportunistic behavior of SC PPP projects have primarily focused on the macro level of influencing factors^25^, management system^26,27^. However, there has been little discussion on the behavior strategy of stakeholders from the micro perspective. In fact, behavior originates from the subjective motivation of human, and the study of decision-making process is key to promoting a better operation of SC projects^28^. Game theory is an effective way to discuss the decision-making process between different interest groups. Kang et al.^29^ adopted bargaining game to analyze the risk sharing and transfer between public and private sectors. Ho and Liu^30^ used game theory to build a claim decision model to help governments identify opportunistic bidding behavior. The above game models are based on the expected utility theory, which is contrary to the assumption of bounded rationality of human. To explore the mechanism of human decision-making, Kahneman and Tversky^31^ proposed Prospect Theory from a psychological perspective. This theory presented the different psychological perceptions of participants in the face of gains and loss. Thaler^32^ also carried out research on decision-making process and put forward Mental Accounting. He pointed out that when people make choices, they internally build mental accounting and the final behavioral decisions are driven by perceived value from it. Thus, Prospect Theory and Mental Accounting both can be applied to the study of human risky behavior. Therefore, this paper explores the evolution mechanism of opportunistic behavior of SC PPP projects from the perspective of perceived value, and analyzes the evolutionary conditions and trend of main stakeholders under different conditions.

The main practical and academic contributions of this paper are as follows: (1) This paper firstly established the perceived value game matrix based on Prospect Theory and Mental Accounting to study the opportunistic behavior in SC PPP projects. From the micro-psychological level, mental accounting and probability function are constructed according to the different preferences of incomes and costs. (2) Different from the previous binary game between public and private two sides, a tripartite game model including private companies, citizens and the government is constructed on account of the existing literature of the importance of the public^21,22^. (3) Through the case of Nanganqu project, the numerical simulation of the constructed model is carried out to verify the validity and feasibility of this model. This paper studies how to realize the effective governance of opportunistic behavior in SC PPP project, and provides new insights for guidance and management of sustainable urban water management.

## Methods

### Application of evolutionary game theory

Evolutionary game theory is a method to analyze the dynamic evolution between groups under the assumption of bounded rationality, which has been widely used in the economic management fields^33^. The behavior of decision makers is not completely rational because of the limited information source and processing ability^34^. Bounded rationality emphasizes that the players adjust and improve their strategies in continuous information interaction and learning, and finally choose the satisfied and optimal strategy^35^. Scholars had used evolutionary game to solve various field problems, including management^36^, environment^37^, and economics^38^. Thus, evolutionary game theory is a suitable method applied to the study of conflict coordination and interaction models between stakeholders. Wang et al.^39^ and Ding et al.^12^ pointed out that the research on stakeholders of SC projects is a topic worthy of further study. Therefore, this paper established evolutionary game model to discuss the problem of opportunistic behavior between the key stakeholders during the construction of SC projects.

Gino and Margolis^40^ acknowledged the complexity of human subjective decision-making. The perceived value of behavioral outcomes varies from person to person due to differences in risk preferences and cognition, which in turn result in different strategic choices. Thus, the existing game payment matrix is still imperfect and needs to be strengthened. It needs to further explore the psychological mechanism of opportunistic behavior from the micro level. Thus, the perceived value game model between main stakeholders is of great significance to the study of opportunistic behavior in SC PPP projects. Therefore, this paper constructs the evolutionary game model from the perspective of perceived value to fill the present research gap.

### Perceived value based on prospect theory and mental accounting

Kahneman and Tversky^31^ put forward Prospect Theory, which held that when people face risky behavior decisions, they are not based on the objective utility of the expected value, but the perceived value. The perceived utility value $U$ is composed of utility function $u(\Delta w)$ and weight function $\pi (p)$. $p$ denotes the probability of an event, $0 \le p \le 1$. $\Delta w$ represents the difference between the result and the expectation.

1 $$U = \sum {u(\Delta w)\pi (p)}$$

Thaler^32^ proposed Mental Accounting, and claimed that people mentally divide the cost and income into different accounts. Influenced by the perceived value of accounts, decision makers may choose different strategies when faced with the same scenario due to different risk attitudes. Based on Prospect Theory and Mental Accounting, this paper constructs the perceived value function of mental accounting. The utility function $u(\Delta w)$ is divided into cost account function $u_{C} (w)$ and income account function $u_{I} (w)$. The details are as follows:

2 $$\begin{aligned} u_{C} (w) & = \left\{ \begin{gathered} \delta (w - E_{0} )^{\tau } ,w \ge E_{0} \hfill \\ - (E_{0} - w)^{\kappa } ,w < E_{0} \hfill \\ \end{gathered} \right. \\ u_{I} (w) & = \left\{ \begin{gathered} (w - E_{1} )^{\chi } ,w \ge E_{1} \hfill \\ - \gamma (E_{1} - w)^{\zeta } ,w < E_{1} \hfill \\ \end{gathered} \right. \\ \end{aligned}$$

**Figure 4:**
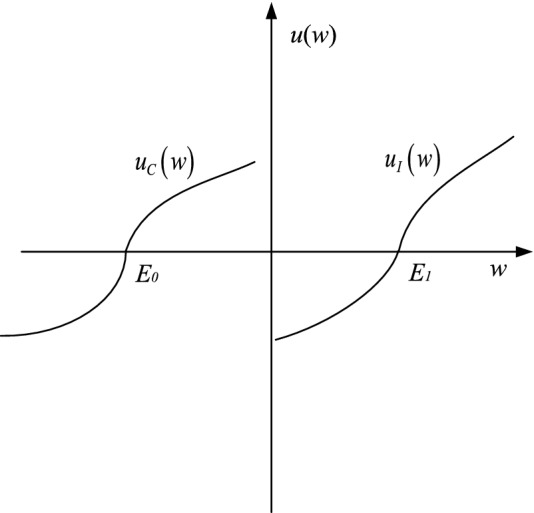
The value functions of cost and income mental accounting.

$E_{0}$ is the expected reference value of cost. $\delta$ represents cost sensitivity coefficient. The higher the value of $\delta$, the more sensitive the decision-maker to cost. $\tau$ and $\kappa$ denotes risk preference coefficient of cost account ($0 < \tau < 1$,$0 < \kappa < 1$). $E_{1}$ is the expected reference value of income. $\gamma$ is defined as income sensitivity coefficient. With the increase of $\gamma$, decision makers are more sensitive to income. $\chi$ and $\zeta$ represent risk preference coefficient of income account ($0 < \chi < 1$,$0 < \zeta < 1$). The figure of function $u_{C} (w)$ and function $u_{I} (w)$ is shown in Fig. 4.

The decision weight function $\pi (p)$ depends on the tendency of the decision maker to choose strategy and is a monotonously increasing function of probability evaluation.

3 $$\pi (p) = \frac{{p^{\beta } }}{{\left( {p^{\beta } + (1 - p)^{\beta } } \right)^{{\frac{1}{\beta }}} }}$$

**Figure 5:**
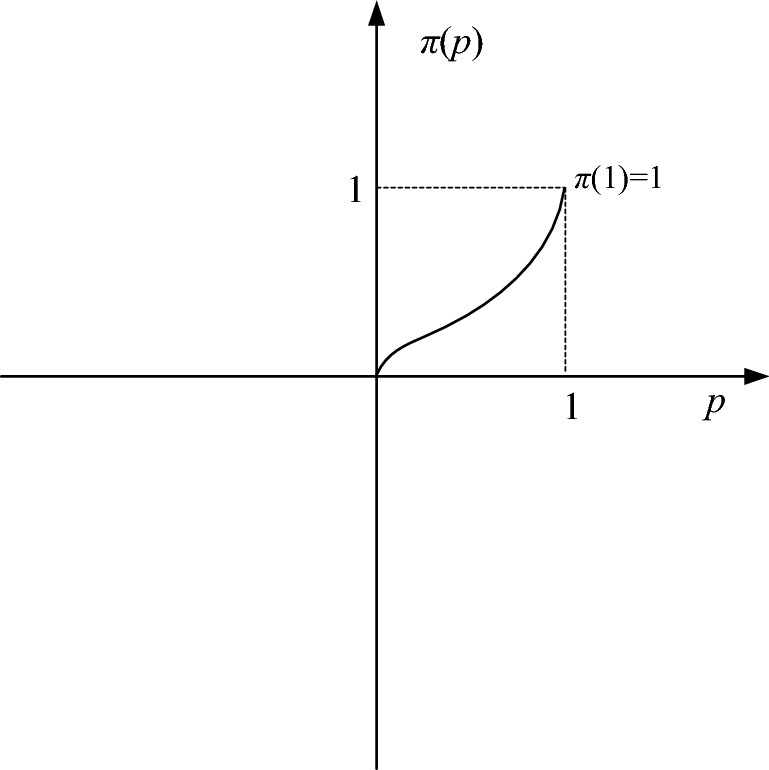
The weigh function of mental accounting.

$\beta$ is decision influence coefficient ($0 < \beta < 1$), the value of $\beta$ depends on the situation. As the value of $\beta$ increases, the decision weight function becomes more curved. The figure of $\pi (p)$ is shown in Fig. 5.

## Tripartite evolutionary game model from the perspective of perceived value

### Problem description and model setup

In the SC PPP project, the government and private companies sign PPP principal-agent contracts to jointly participate in the construction of SC. According to the contract, the government grants management authority to private companies, which are responsible for construction and operation. However, the private companies, as the direct beneficiary of opportunistic behavior, are more likely to have moral hazard behavior in project construction^23^. As users and potential payers of the SP projects, the public can express their views through formal or informal channels, so as to supervise the project and safeguard their interest^21^. Previous literature has proved that the power of public supervision cannot be underestimated^41^. As the initiator of the SC project, the government’s attitude greatly influences the construction quality and the behavior of other participants^42^.

**Figure 6:**
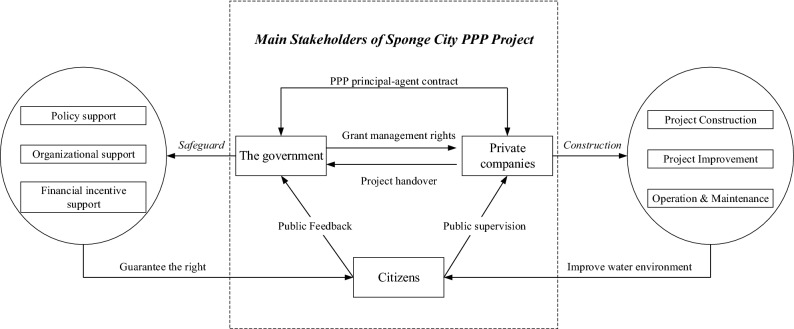
Main stakeholders of Sponge City PPP project.

In the 1980s, Freeman^43^ introduced the concept of stakeholders in the context of company management and defined stakeholders as “groups or individuals vital to the survival of the organization”. Since then, this concept has been widely accepted^44^. In this paper, stakeholders are defined as the groups that can influence the efficiency, effectiveness, and effect of project implementation, and they can obtain personal perceived benefits (gains or losses) from the SC projects. Based on the previous analysis, the main stakeholders of this paper are private companies, citizens and the government. The relationship between main stakeholders is shown in Fig. 6.

The three parties are designed as the large and finite populations of the selected pure strategy. Relevant assumptions are as follows:

#### **Assumption 1**

There are only three participants in the game model of opportunistic behavior of Sponge City PPP projects, namely, private companies, citizens and the government, and all of them are bounded rational. The choice of their strategies is mainly based on their perceived value of the income and cost of the strategy, not the value of the strategy itself.

#### **Assumption 2**

The strategy set of private companies is {P1, P2}. Private companies can choose strategy P1 to make efforts to build SC projects, such as seriously promoting project construction and enhancing construction capacity. Strategy P2 represents the choice of opportunistic behavior, to obtain excess profits through moral hazard behavior. The proportion that private companies choose strategy P1 is $x \in [0,1][0,1]$, the proportion of strategy P2, in accordance, becomes $1 - x$.

#### **Assumption 3**

The strategy set of citizens is {C1, C2}. Citizens, as beneficiaries and users of SC projects, can choose the strategy C1 to participate in regulation by reporting and disclosing opportunistic behavior. There is also a choice to adopt no supervision strategy C2. The proportion of the above two strategy choices is expressed as $y \in [0,1]$ and $1 - y$, separately.

#### **Assumption 4**

The government has two pure strategic choices: it may select to strict regulation strategy G1, such as conducting strict supervision and scrutiny for each phase of the project, as well as follow up the project process at all time, or elect to pursue loose regulation strategy G2 to reduce the workload. The strategy set is {G1, G2}, denoted by $z \in [0,1]$ and $1 - z$, respectively.

### Model parameters and perceived payoff function

**Figure 7:**
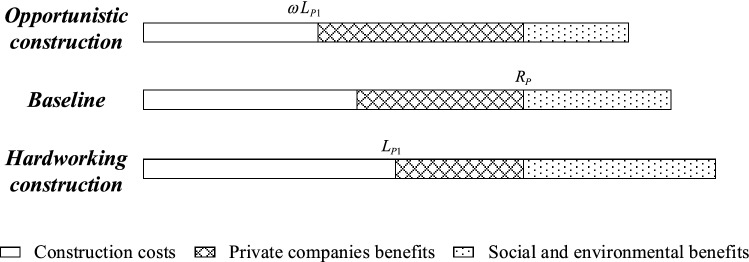
Cost–benefit diagram of opportunistic and hardworking construction.

As shown in Fig. 7, opportunistic behavior brings considerable benefits to private companies at the expense of the interests of the government and public^15,45^. Based on Section 2 literature review, the model parameters are set as follows.

Before the launch of SC PPP projects, the government and private companies negotiate the total construction investment $R_{P}$. For the private companies, the construction cost under the effort construction strategy P1 is $L_{P1}$. $\omega L_{P1}$ denotes the construction cost under strategy P2, $\omega$ is defined as the opportunism cost discount coefficient $(0 < \omega < 1)$. Under strict government supervision, opportunism company disclosure by the citizens is fined $F$. Meanwhile, companies need to take rectification and other measures to compensate the public with the value of $E_{P}$. $S_{P}$ represents the reputation loss of companies caused by citizens participation in the supervision and disclosure of opportunistic behavior under the loose government supervision.

For citizens, the benefit of the good living environment brought by the construction of SC is $H_{{{\text{P}}1}}$. Under opportunistic construction, the ecological benefit to citizens is $\eta H_{P1}$, where $\eta$ is the benefit discount coefficient $(0 < \eta < 1)$. $C_{C1}$ denotes the cost to regulate opportunistic companies. $E_{G}$ represents the material and moral rewards that citizens receive for revealing the opportunistic behavior under strict government regulation.

For the government, the ecological and social income obtained under strict supervision is $G_{G1}$. $C_{G}$ represents the additional supervision cost of handling public reports. Under the loose supervision strategy G2, the incomes obtained are recorded as $G_{G2}$. $S_{G}$ denotes the cost of the government’s reputation loss caused by the public reporting opportunistic construction under the loose regulation strategy G2. The specifications of the parameters are further described in Table 1.

**Table 1:** Parameter definitions of tripartite evolutionary game model.

Parameters	Description
$R_{P}$	The total construction investment of SC PPP project
$L_{P1}$	The construction cost when private companies choose strategy P1
$S_{P}$	The reputation loss for private companies when opportunistic behavior is revealed
$F$	Fines of opportunistic behavior for private companies
$E_{P}$	The compensation for citizens from private companies
$H_{{{\text{P}}1}}$	The ecological income for citizens when private companies choose strategy P1
$C_{C1}$	The cost for citizens when choosing strategy C1
$E_{G}$	The rewards income of citizens for revealing under strategy G1
$G_{G1}$	The income when the government choose strategy G1
$G_{G2}$	The income when the government choose strategy G2
$C_{G}$	The additional supervision cost for the government to handle public reports
$S_{G}$	The reputation loss for government when opportunistic behavior is revealed
$\omega$	Discount coefficient of cost under opportunistic construction $(0 < \omega < 1)$
$\eta$	Discount coefficient of income under opportunistic construction $(0 < \eta < 1)$

**Figure 8:**
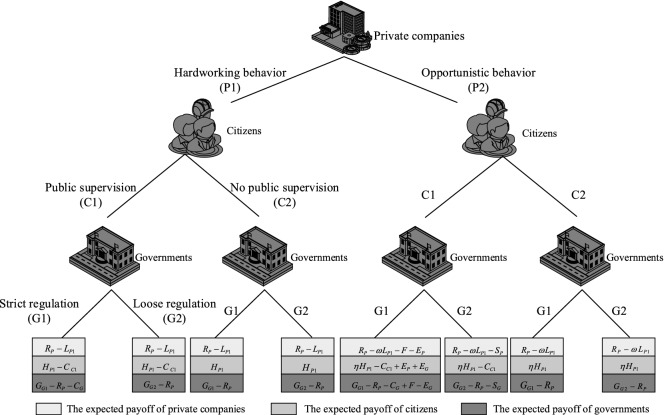
Game tree and traditional expected payoff.

According to assumptions and parameter descriptions, the game tree and traditional expected payoff of main stakeholders are constructed, as shown in Fig. 8. The game tree intuitively describes the interactive interest relationship between the private companies, citizens and the government.

However, the traditional expected payoff is based on the objective utility value and does not fully take into account the subjective evaluation and risk attitude of the participants. Therefore, this paper optimizes and improves the game matrix in combination with Prospect Theory and Mental Accounting. The perceived payoff matrix is shown in Table 2.

**Table 2:** Perceived payoff matrix based on Prospect Theory and Mental Accounting.

	The government G1	The government G2
**Private companies P1**		
Citizens C1	P1: $u_{I} (R_{P} ) - u_{C} (L_{P1} )$	P1: $u_{I} (R_{P} ) - u_{C} (L_{P1} )$
	C1: $u_{I} (H_{P1} ) - u_{C} (C{}_{C1})$	C1: $u_{I} (H_{P1} ) - u_{C} (C{}_{C1})$
	G1: $u_{I} (G_{G1} ) - u_{C} (R_{P} + C_{G} )$	G2: $u_{I} (G_{G2} ) - u_{C} (R_{P} )$
Citizens C2	P1: $u_{I} (R_{P} ) - u_{C} (L_{P1} )$	P1: $u_{I} (R_{P} ) - u_{C} (L_{P1} )$
	C2: $u_{I} (H_{P1} )$	C2: $u_{I} (H_{P1} )$
	G1: $u_{I} (G_{G1} ) - u_{C} (R_{P} )$	G2: $u_{I} (G_{G2} ) - u_{C} (R_{P} )$
**Private companies P2**		
Citizens C1	P2: $u_{I} (R_{P} ) - u_{C} (\omega L_{P1} + F + E_{P} )$	P2: $u_{I} (R_{P} ) - u_{C} (\omega L_{P1} + S_{P} )$
	C1: $u_{I} (\eta H_{P1} + E_{P} + E_{G} ) - u_{C} (C_{C1} )$	C1: $u_{I} (\eta H_{P1} ) - u_{C} (C_{C1} )$
	G1: $u_{I} (G_{G1} + F) - u_{C} (R_{P} + C_{G} + E_{G} )$	G2: $u_{I} (G_{G2} ) - u_{C} (R_{P} + S_{G} )$
Citizens C2	P2: $u_{I} (R_{P} ) - u_{C} (\omega L_{P1} )$	P2: $u_{I} (R_{P} ) - u_{C} (\omega L_{P1} )$
	C2: $u_{I} (\eta H_{P1} )$	C2: $u_{I} (\eta H_{P1} )$
	G1: $u_{I} (G_{G1} ) - u_{C} (R_{P} )$	G2: $u_{I} (G_{G2} ) - u_{C} (R_{P} )$

### Replicated dynamic equations of tripartite evolutionary game

According to the above model assumptions and perceived payoff matrix, the perceived utility functions of private companies, citizens and government can be construceted respectively (see further details in Appendix A). Friedman^43^ claimed that when the return of a strategy in the game is higher than the average return of other strategies, then this strategy can have strong resistance to prevent the invasion of mutation strategy, so as to drive the adaptation of the group evolution process. Replicated dynamics equations of private companies, citizens and the government are solved respectively.

We supposed $V_{P1}$ and $V_{P2}$ to represent the perceived utility of private companies for selecting strategy P1 and strategy P2, respectively. And $\overline{V}_{P}$ represents the expected perceived utility of private companies. Then, the consequent replicated dynamic equation of private companies adopting strategy P1 can be described as follows:

4 $$\begin{aligned} F(x) & = \frac{dx}{{dt}} = x(V_{P1} - \overline{V}_{P} ) = x(1 - x)(V_{P1} - V_{P2} ) \\ & = x(1 - x)\left[ {\pi (y)\pi (z)\left[ {u_{I} (R_{p} ) - u_{I} (R_{p} ) - u_{C} (L_{P1} ) + u_{C} (\omega L_{P1} + F + E_{P} )} \right]} \right. \\ & \quad + \pi (y)\pi (1 - z)\left[ {u_{I} (R_{p} ) - u_{I} (R_{p} ) - u_{C} (L_{P1} ) + u_{C} (\omega L_{P1} + S_{P} )} \right] \\ & \quad + \pi (1 - y)\pi (z)\left[ {u_{I} (R_{p} ) - u_{I} (R_{p} ) - u_{C} (L_{P1} ) + u_{C} (\omega L_{P1} )} \right] \\ & \quad + \pi (1 - y)\pi (1 - z)\left[ {u_{I} (R_{p} ) - u_{I} (R_{p} ) - u_{C} (L_{P1} ) + u_{C} (\omega L_{P1} )} \right] \\ \end{aligned}$$

Similarly, we let $V_{C1}$ and $V_{C2}$ denote the perceived utility of citizens for choosing strategy C1 and strategy C2, respectively. The expected perceived utility of citizens is defined as $\overline{V}_{C}$. Then, the replicated dynamic equation of citizens adopting strategy C1 can be formulated as follows:

5 $$\begin{aligned} F(y) & = \frac{dy}{{dt}} = y(V_{C1} - \overline{V}_{C} ) = y(1 - y)(V_{C1} - V_{C2} ) \\ & = y(1 - y)\left[ {\pi (x)\pi (z)\left[ {u_{I} (H_{P1} ) - u_{I} (H_{P1} ) - u_{C} (C{}_{C1})} \right]} \right. \\ & \quad + \pi (x)\pi (1 - z)\left[ {u_{I} (H_{P1} ) - u_{I} (H_{P1} ) - u_{C} (C{}_{C1})} \right] \\ & \quad + \pi (1 - x)\pi (z)\left[ {u_{I} (E_{P} + E_{G} + \eta H_{P1} ) - u_{I} (\eta H_{P1} ) - u_{C} (C_{C1} )} \right] \\ & \quad + \pi (1 - x)\pi (1 - z)\left[ {u_{I} (\eta H_{P1} ) - u_{I} (\eta H_{P1} ) - u_{C} (C_{C1} )} \right] \\ \end{aligned}$$

Accordingly, $V_{G1}$ and $V_{G2}$ signify the perceived utility of the governments for choosing strategy G1 and strategy G2, respectively. And $\overline{V}_{G}$ represents the expected perceived utility of the government. Then, the replicated dynamic equation of the government adopting strategy G1 is defined as follows:

6 $$\begin{aligned} F(z) & = \frac{dz}{{dt}} = z(V_{G1} - \overline{V}_{G} ) = z(1 - z)(V_{G1} - V_{G2} ) \\ & = z(1 - z)\left[ {\pi (x)\pi (y)\left[ {u_{I} (G_{G1} ) - u_{I} (G_{G2} ) - u_{C} (R_{P} + C_{G} ) + u_{C} (R_{P} )} \right]} \right. \\ & \quad + \pi (x)\pi (1 - y)\left[ {u_{I} (G_{G1} ) - u_{I} (G_{G2} ) - u_{C} (R_{P} ) + u_{C} (R_{P} )} \right] \\ & \quad + \pi (1 - x)\pi (y)\left[ {u_{I} (G_{G1} + F) - u_{I} (G_{G2} ) - u_{C} (R_{P} + C_{G} + E_{G} ) + u_{C} (R_{P} + S_{G} )} \right] \\ & \quad + \pi (1 - x)\pi (1 - y)\left[ {u_{I} (G_{G1} ) - u_{I} (G_{G2} ) - u_{C} \left( {R_{P} + (1 - \omega )L_{P1} } \right) + u_{C} \left( {R_{P} + (1 - \omega )L_{P1} } \right)} \right] \\ \end{aligned}$$

Equations (4)–(6) constitute a tripartite replicated dynamic system with private companies, citizens, and government as main stakeholders. The strategy of each stakeholder changes over time in response to the evolution of other game participants’ choices. This evolution process is explained by the fact that during the evolutionary game, the game participants consciously change their strategies through learning mechanisms to maximize their personal interests. Thus, we further conducted the model analysis to explore the law of these stakeholders’ strategy choices.

## Model analysis

### Strategy stability analysis of each participant

In evolutionary game, three players dynamically adjust their strategies by adjusting the values of $x$, $y$ and $z$, and optimize their strategies through learning and imitation. Evolutionary stable strategy (ESS) points must be robust to minor disturbances according to the stability theorem of differential equations and the properties of ESS^36^. In order to clarify the strategic choice of each participant, the dynamic evolution trend of three players is analyzed respectively, therefore, the critical condition for decision-making is identified.

#### Strategy stability analysis of private companies

The probability that private companies choose strategy P1 is $x$. According to Eq. (4), the first derivative of $F(x)$ is as follows:

7 $$\frac{dF(x)}{{dx}} = (1 - 2x)\left[ {u_{C} (\omega L_{P1} - L_{P1} ) + \pi (y)\pi (z)u_{C} (F + E_{p} ) + \pi (y)\pi (1 - z)u_{C} (S_{P} )} \right]$$

When $\pi (y) = \pi (y)* = \frac{{u_{C} (L_{P1} - \omega L_{P1} )}}{{\pi (z)u_{C} (F + E_{p} ) + \pi (1 - z)u_{C} (S_{P} )}}$, then $\frac{dF(x)}{{dx}} \equiv 0$. It indicates that all strategies of private companies are at a steady state. Otherwise, when $\pi (y) \ne \pi (y)*$, if under the condition that $\pi (y) < \pi (y)*$, the results come as follows: $\frac{dF(x)}{{dx}}|_{x = 0} < 0$, $\frac{dF(x)}{{dx}}|_{x = 1} > 0$. In this case, it indicates that $x = 0$ is ESS and private companies tend to choose strategy P2. If under the condition that $\pi (y) > \pi (y)*$, then, $\frac{dF(x)}{{dx}}|_{x = 0} > 0$, $\frac{dF(x)}{{dx}}|_{x = 1} < 0$. In this case, it implies that $x = 1$ is ESS and private companies prefer to choose strategy P1. Therefore, the response function of the probability $x$ that private companies choose hardworking behavior strategy P1 is as follows:

8 $$x = \left\{ {\begin{array}{*{20}l} {0,} \hfill & {if\;\pi (y) < \frac{{u_{C} (L_{P1} - \omega L_{P1} )}}{{\pi (z)u_{C} (F + E_{p} ) + \pi (1 - z)u_{C} (S_{P} )}}} \hfill \\ {[0,1],} \hfill & {if\;\pi (y) = \frac{{u_{C} (L_{P1} - \omega L_{P1} )}}{{\pi (z)u_{C} (F + E_{p} ) + \pi (1 - z)u_{C} (S_{P} )}}} \hfill \\ {1,} \hfill & {if\;\pi (y) > \frac{{u_{C} (L_{P1} - \omega L_{P1} )}}{{\pi (z)u_{C} (F + E_{p} ) + \pi (1 - z)u_{C} (S_{P} )}}} \hfill \\ \end{array} } \right.$$

**Figure 9:**
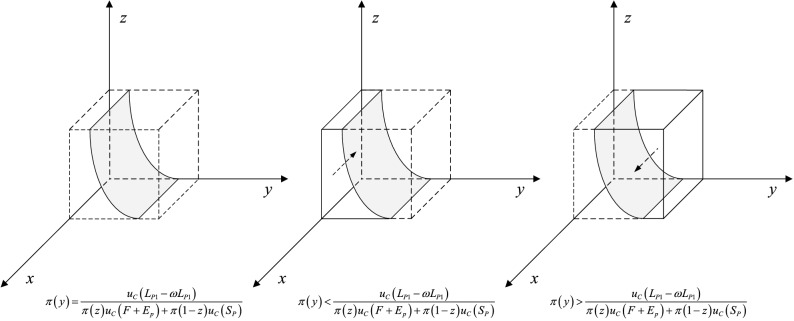
Replicated dynamic phase diagram of private company.

The dynamic phase diagram of private company is accordingly shown in Fig. 9. As shown above, the evolutionary equilibrium state of private companies is closely related to the strategy choice of citizens and the government. Along with the increase in the probability of citizens participation in regulation, the probability of private companies adopting effortful behavior strategy P1 increases and gradually converges to 1. Further analysis of the model shows that, in order to drive private companies to choose the P1 strategy, increasing the perceived value of government penalty $u_{C} (F)$ and reputation loss $u_{C} (S_{P} )$ is an effective method.

#### Strategy stability analysis of citizens

The probability that citizens choose strategy C1 is $y$. According to Eq. (5), the first derivative of $F(y)$ is as follows:

9 $$\frac{dF(y)}{{dy}} = (1 - 2y)\left[ { - u_{C} (C_{C1} ) + \pi (1 - x)\pi (z)u_{I} (E_{G} + E_{p} )} \right]$$

When $\pi (z) = \pi (z)* = \frac{{u_{C} (C_{C1} )}}{{\pi (1 - x)u_{I} (E_{G} + E_{p} )}}$, then $\frac{dF(y)}{{dy}} \equiv 0$. It implies that all strategies of citizens are at a steady state. Otherwise, when $\pi (z) \ne \pi (z)*$, if under the condition that $\pi (z) < \pi (z)*$, the results come as follows: $\frac{dF(y)}{{dy}}|_{y = 0} < 0$, $\frac{dF(y)}{{dy}}|_{y = 1} > 0$. In this case, it represents that $y = 0$ is ESS and citizens prefer to choose strategy C2. If under the condition that $\pi (z) > \pi (z)*$, then, $\frac{dF(y)}{{dy}}|_{y = 0} > 0$, $\frac{dF(y)}{{dy}}|_{y = 1} < 0$. In this case, it implies that $y = 1$ is ESS and citizens incline to choose strategy C1. Therefore, the response function of the probability $y$ that citizens choose to impose supervision strategy C1 is as follows:

10 $$y = \left\{ {\begin{array}{*{20}l} 0 \hfill & {if\;\pi (z) < \frac{{u_{C} (C_{C1} )}}{{\pi (1 - x)u_{I} (E_{G} + E_{p} )}}} \hfill \\ {[0,1]} \hfill & {if\;\pi (z) = \frac{{u_{C} (C_{C1} )}}{{\pi (1 - x)u_{I} (E_{G} + E_{p} )}}} \hfill \\ 1 \hfill & {if\;\pi (z) > \frac{{u_{C} (C_{C1} )}}{{\pi (1 - x)u_{I} (E_{G} + E_{p} )}}} \hfill \\ \end{array} } \right.$$

**Figure 10:**
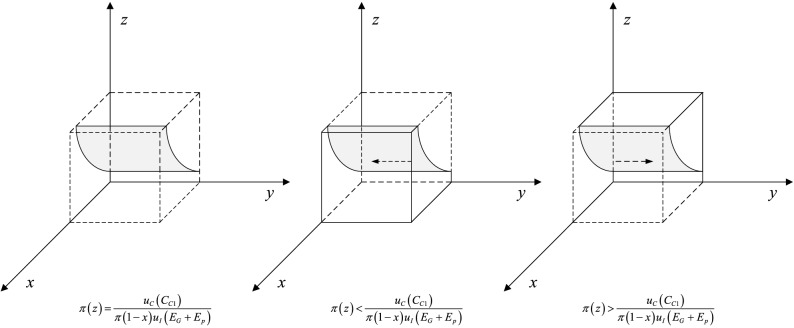
Replicated dynamic phase diagram of citizen.

The dynamic phase diagram of citizen is accordingly shown in Fig. 10. The evolutionary equilibrium of citizens is closely related to the strategic choice of private companies and the government. With the increase of the probability of strict government supervision, the probability of strategy C1 that citizens participate in supervision increases and converges to 1. In addition, it is an effective way to increase the perceived value of compensation $u_{I} (E_{G} )$ and $u_{I} (E_{P} )$ to motivate the public to participate in supervision.

#### Strategy stability analysis of the government

The probability that the government chooses strategy G1 is $z$. According to Eq. (6), the first derivative of $F(z)$ is as follows:

11 $$\frac{dF(z)}{{dz}} = (1 - 2z)\left[ {u_{I} (G_{G1} - G_{G2} ) + \pi (y)\left[ { - u_{C} (C_{G} ) + \pi (1 - x)\left[ {u_{I} (F) + u_{C} (S_{G} - E_{G} )} \right]} \right]} \right]$$

When $\pi (x) = \pi (x)* = \frac{{\pi (y)\left[ {u_{I} (F) + u_{C} (S_{G} - C_{G} - E_{G} )} \right] + u_{I} (G_{G1} - G_{G2} )}}{{\pi (y)\left[ {u_{I} (F) + u_{C} (S_{G} - E_{G} )} \right]}}$, then $\frac{dF(z)}{{dz}} \equiv 0$. It implies that all strategies of the government are at a steady state. Otherwise, when $\pi (x) \ne \pi (x)*$, if under the condition that $\pi (x) < \pi (x)*$, the results come as follows: $\frac{dF(z)}{{dz}}|_{z = 0} > 0$, $\frac{dF(z)}{{dz}}|_{z = 1} < 0$. In this case, it represents that $z = 1$ is ESS and the government tends to choose strategy G1 to implement strict regulation. If under the condition that $\pi (x) > \pi (x)*$, then, $\frac{dF(z)}{{dz}}|_{z = 0} < 0$, $\frac{dF(z)}{{dz}}|_{z = 1} > 0$. In this case, it implies that $z = 0$ is ESS and the government inclines to choose strategy G2. Therefore, the response function of the probability $z$ that the government select strict supervision strategy G1 is as follows:

12 $$z = \left\{ {\begin{array}{*{20}l} 0 \hfill & {if\;\pi (x) > \frac{{\pi (y)\left[ {u_{I} (F) + u_{C} (S_{G} - C_{G} - E_{G} )} \right] + u_{I} (G_{G1} - G_{G2} )}}{{\pi (y)\left[ {u_{I} (F) + u_{C} (S_{G} - E_{G} )} \right]}}} \hfill \\ {[0,1]} \hfill & {if\;\pi (x) = \frac{{\pi (y)\left[ {u_{I} (F) + u_{C} (S_{G} - C_{G} - E_{G} )} \right] + u_{I} (G_{G1} - G_{G2} )}}{{\pi (y)\left[ {u_{I} (F) + u_{C} (S_{G} - E_{G} )} \right]}}} \hfill \\ 1 \hfill & {if\;\pi (x) < \frac{{\pi (y)\left[ {u_{I} (F) + u_{C} (S_{G} - C_{G} - E_{G} )} \right] + u_{I} (G_{G1} - G_{G2} )}}{{\pi (y)\left[ {u_{I} (F) + u_{C} (S_{G} - E_{G} )} \right]}}} \hfill \\ \end{array} } \right.$$

**Figure 11:**
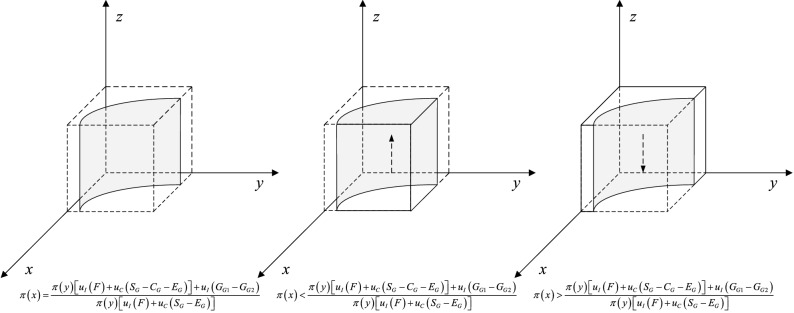
Replicated dynamic phase diagram of the government.

The dynamic phase diagram of the government is accordingly shown in Fig. 11. The evolutionary equilibrium state of the government is closely related to the strategic choices of private companies and citizens. Along with the increase of the probability of private companies choosing strategy P2, the probability of strict regulation strategy G1 that increases and converges to 1. Further analysis based on the response function reveals that increasing the perceived benefits under strict supervision can promote positive regulatory behavior.

### Strategy stability analysis of tripartite evolutionary game system

In combination with Eqs. (4), (5) and (6), a tripartite evolutionary dynamic system is obtained as follows:

13 $$\left\{ {\begin{array}{*{20}l} {F(x) = \frac{dx}{{dt}} = x(1 - x)(V_{P1} - V_{P2} ) = 0} \hfill \\ {F(y) = \frac{dy}{{dt}} = y(1 - y)(V_{C1} - V_{C2} ) = 0} \hfill \\ {F(z) = \frac{dz}{{dt}} = z(1 - z)(V_{G1} - V_{G2} ) = 0} \hfill \\ \end{array} } \right.$$

Solving the equations, 8 pure strategy equilibrium points are obtained, as $P_{1} (0,0,0)$, $P_{2} (1,0,0)$, $P_{3} (0,1,0)$, $P_{4} (0,0,1)$, $P_{5} (1,1,0)$, $P_{6} (1,0,1)$, $P_{7} (0,1,1)$, $P_{8} (1,1,1)$. According to the Lyapunov stability theory, the equilibrium of the replicated dynamic system is stable only if the eigenvalues of the Jacobian have negative real parts^33^. Specifically, when the eigenvalues of the Jacobian matrix corresponding to an equilibrium point are negative, the equilibrium point is a local stable point. While at least one eigenvalue of the Jacobian matrix is positive, the point is an unstable point. The Jacobian matrix of the system (13) can be obtained as:

14 $$J = \left[ \begin{gathered} J_{11} J_{12} J_{13} \hfill \\ J_{21} J_{22} J_{23} \hfill \\ J_{31} J_{32} J_{33} \hfill \\ \end{gathered} \right]$$

15 $$\left\{ {\begin{array}{*{20}l} {J_{11} = \frac{\partial F(x)}{{\partial x}} = (1 - 2x)\left[ {u_{L} (\omega L_{P1} - L_{P1} ) + \pi (y)\pi (z)u_{L} (F + E_{p} ) + \pi (y)\pi (1 - z)u_{L} (S_{P} )} \right]} \hfill \\ {J_{12} = \frac{\partial F(x)}{{\partial y}} = x(1 - x)\left[ {\pi (z)u_{L} (F + E_{p} ) + \pi (1 - z)u_{L} (S_{P} )} \right]} \hfill \\ {J_{13} = \frac{\partial F(x)}{{\partial z}} = x(1 - x)\left[ {\pi (y)u_{L} (F + E_{p} ) - \pi (y)u_{L} (S_{P} )} \right]} \hfill \\ {J_{21} = \frac{\partial F(y)}{{\partial x}} = y(1 - y)\left[ { - \pi (z)u_{G} (E_{G} + E_{p} )} \right]} \hfill \\ {J_{22} = \frac{\partial F(y)}{{\partial y}} = (1 - 2y)\left[ { - u_{L} (C_{C1} ) + \pi (1 - x)\pi (z)u_{G} (E_{G} + E_{p} )} \right]} \hfill \\ {J_{23} = \frac{\partial F(y)}{{\partial z}} = y(1 - y)\left[ {\pi (1 - x)u_{G} (E_{G} + E_{p} )} \right]} \hfill \\ {J_{31} = \frac{\partial F(z)}{{\partial x}} = z(1 - z)\left[ { - \pi (y)\left[ {u_{G} (F) + u_{L} (S_{G} - E_{G} )} \right]} \right]} \hfill \\ {J_{32} = \frac{\partial F(z)}{{\partial y}} = z(1 - z)\left[ { - u_{L} (C_{G} ) + \pi (1 - x)\left[ {u_{G} (F) + u_{L} (S_{G} - E_{G} )} \right]} \right]} \hfill \\ {J_{33} = \frac{\partial F(z)}{{\partial z}} = (1 - 2z)\left[ {u_{G} (G_{G1} - G_{G2} ) + \pi (y)\left[ { - u_{L} (C_{G} ) + \pi (1 - x)\left[ {u_{G} (F) + u_{L} (S_{G} - E_{G} )} \right]} \right]} \right]} \hfill \\ \end{array} } \right.$$

As shown in Table 3, since the cost of public supervision is greater than 0, that is, $u_{C} (C_{C1} ) > 0$, then it is found that eigenvalue $\lambda_{{{ 2}}} > 0$ of $P_{3} (0,1,0)$, $P_{5} (1,1,0)$ and $P_{8} (1,1,1)$. It is obvious that benefits obtained under strict government supervision are higher than that of loose supervision. Due to $u_{I} (G_{G1} - G_{G2} ) > 0$, as a result eigenvalue $\lambda_{{{ 3}}} > 0$ in $P_{1} (0,0,0)$ and $P_{2} (1,0,0)$. As for eigenvalue $\lambda_{{{ 2}}}$ of $P_{4} (0,0,1)$, the reward and compensation from reporting opportunistic behavior is greater than the cost. Then it can be found that $u_{I} (E_{G} + E_{p} ) > u_{C} (C_{C1} )$, thus, $P_{4} (0,0,1)$ is not an equilibrium point. For $u_{C} (L_{P1} - \omega L_{P1} ) > 0$, therefore, the eigenvalue $\lambda_{{{ 1}}}$ of $P_{6} (1,0,1)$ is larger than 0. Based on the actual situation, the penalties for opportunistic behavior in private companies outweigh the benefits, that is, $u_{C} (F + E_{p} ) > u_{C} (L_{P1} - \omega L_{P1} )$. Then eigenvalue $\lambda_{{{ 1}}} > 0$ of $P_{7} (0,1,1)$. Therefore, there is no stable equilibrium point in the tripartite game model according to the reality background.

**Table 3:** Equilibrium points and eigenvalues of tripartite evolutionary game model.

Equilibrium points	Eigenvalues		
	$\lambda_{{{ 1}}}$	$\lambda_{{{ 2}}}$	$\lambda_{{{ 3}}}$
$P_{1} \left( {0,0,0} \right)$	$u_{C} \left( {\omega L_{P1} - L_{P1} } \right)$	$u_{C} \left( { - C_{C1} } \right)$	$u_{I} \left( {G_{G1} - G_{G2} } \right)$
$P_{2} \left( {1,0,0} \right)$	$u_{C} \left( {L_{P1} - \omega L_{P1} } \right)$	$u_{C} \left( { - C_{C1} } \right)$	$u_{I} \left( {G_{G1} - G_{G2} } \right)$
$P_{3} \left( {0,1,0} \right)$	$u_{C} \left( {\omega L_{P1} - L_{P1} + S_{P} } \right)$	$u_{C} \left( {C_{C1} } \right)$	$u_{I} \left( {G_{G1} - G_{G2} + F} \right) + u_{C} \left( {S_{G} - E_{G} - C_{G} } \right)$
$P_{4} \left( {0,0,1} \right)$	$u_{C} \left( {\omega L_{P1} - L_{P1} } \right)$	$u_{C} \left( { - C_{C1} } \right) + u\left( {E_{G} + E_{P} } \right)$	$u_{I} \left( {G_{G2} - G_{G1} } \right)$
$P_{5} \left( {1,1,0} \right)$	$u_{C} \left( {L_{P1} - \omega L_{P1} - S_{P} } \right)$	$u_{C} \left( {C_{C1} } \right)$	$u_{I} \left( {G_{G1} - G_{G2} } \right) - u_{C} \left( {C_{G} } \right)$
$P_{6} \left( {1,0,1} \right)$	$u_{C} \left( {L_{P1} - \omega L_{P1} } \right)$	$u_{C} \left( { - C_{C1} } \right)$	$u_{I} \left( {G_{G2} - G_{G1} } \right)$
$P_{7} \left( {0,1,1} \right)$	$u_{C} \left( {\omega L_{P1} - L_{P1} + F + E_{P} } \right)$	$u_{C} \left( {C_{C1} } \right) + u\left( { - E_{G} - E_{P} } \right)$	$u_{I} \left( {G_{G2} - G_{G1} - F} \right) + u_{C} \left( {C_{G} + E_{G} - S_{G} } \right)$
$P_{8} \left( {1,1,1} \right)$	$u_{C} \left( {L_{P1} - \omega L_{P1} - F - E_{P} } \right)$	$u_{C} \left( {C_{C1} } \right)$	$u_{I} \left( {G_{G2} - G_{G1} } \right) + u_{C} \left( {C_{G} } \right)$

## Numerical simulation analysis

**Figure 12:**
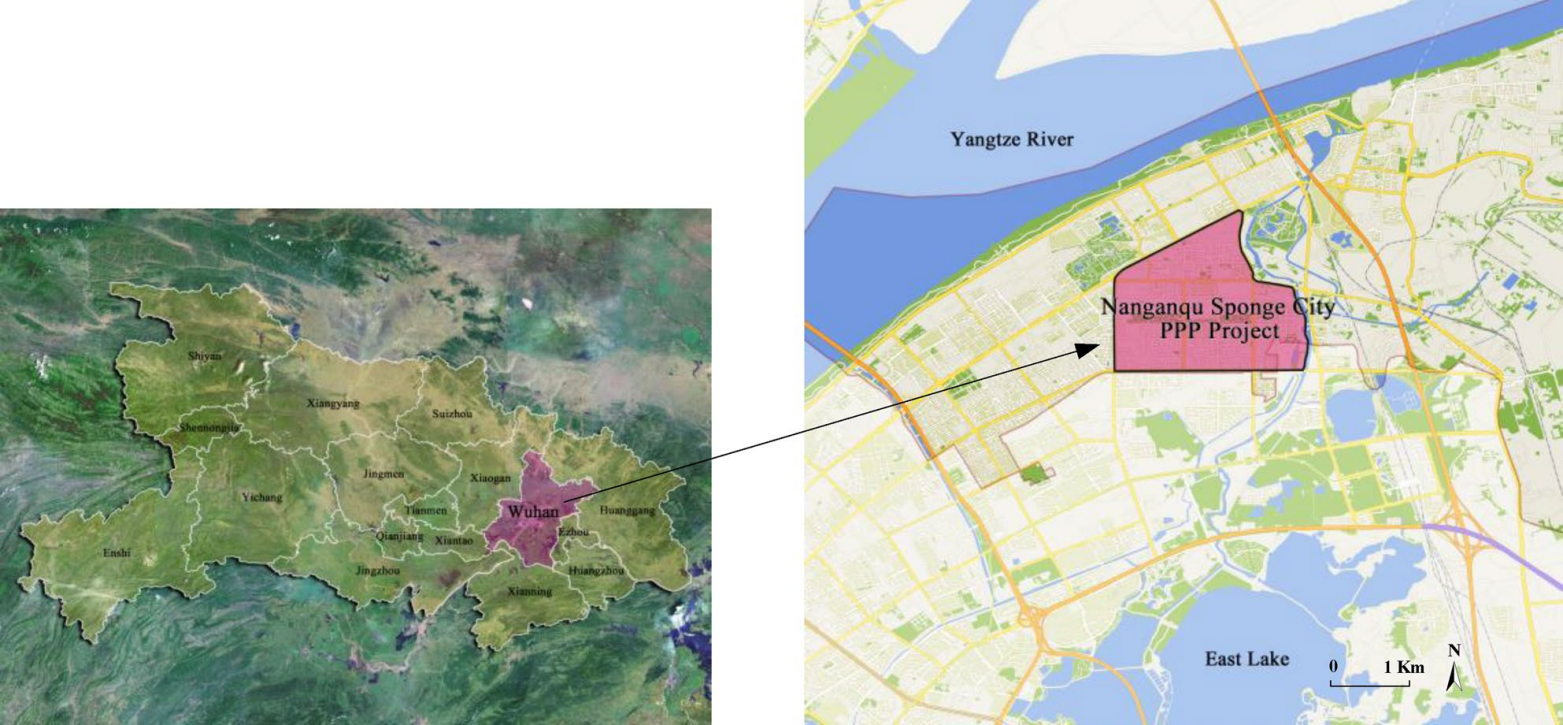
Location of the study area.

Numerical simulation reveals the dynamic evolution of the system which can clarify the changing speed and trend of the participants’ strategies.The parameter setting of numerical simulation in this paper is based on the Nanganqu Project in Qingshan District, Wuhan. Wuhan is a central city in middle-area of China, known as the “city of hundreds of lakes’. Due to its old urban rainwater pipe facilities, it is difficult to load the pressure of urban rainwater drainage, resulting in frequent flood disasters, which have attracted the attention of the Chinese governments. In 2015, Wuhan was selected as one of the first batch of SC pilot cities in China through the competitive evaluation. The government planned to invest 16.29 billion RMB for the construction of SC projects. Among them, Nanganqu project (Fig. 12) is one of the two core demonstration areas that Wuhan has concentrated its efforts on. The total construction area of Nanganqu project is about 3.84 km^2^, involving schools, communities, roads and other public places. This project took detailed consideration of residents’ demands and benefited nearly 100,000 residents in the surrounding area, which is an important people-friendly project. In addition, this project has achieved many practical achievements in reconstruction technology, resource utilization and other aspects, and exported the project experience to provide an effective reference for other cities, which has typical representative significance^46^. Through official document^47^ and literature review, the initial estimated parameter values are set in Table 4. Empirical research showed that the values of relevant parameters in the value function and weight function vary within a certain range^44^. According to the research of Van^49^ and Senbil^48^, the preference coefficients are set as follows: $\tau = 0.88$, $\kappa = 0.88$, $\chi = 0.98$, $\zeta = 0.98$, $\delta = 2$, $\gamma = 2$, $\beta = 0.69$. In order to obviously observe the changing election of behavior trend, the initial probability of $(x,y,z)$ is set to $(0.5,0.5,0.5)$. The results of numerical simulation are analyzed as follows.

**Table 4:** Initial parameter values of tripartite evolutionary game model.

Parameters	Rationale	Value/hundred million RMB
$R_{P}$	According to the official documents, the project cost in the contract is 756 million RMB and other costs of project construction are 121 million RMB. Therefore, the total construction investment $R_{P}$ is set as 8.77 hundred million RMB	8.77
$L_{P1}$	The total construction area of this project is 3.84 km2. Under the hardworking construction strategy P1, we assume that the construction cost per square kilometer is 1.8 hundred RMB, then the value of $L_{P1}$ is 6.912 hundred million RMB	6.912
$S_{P}$	The total population of Wuhan is 12.3 million. Assuming that the reputation value of each person is 10 RMB, so that $S_{P}$ is estimated to be 1.23 hundred million RMB	1.23
$F$	Government penalty $F$ for opportunistic behavior by private companies is assumed as 10% of the contract price, then the value of $F$ is 0.877 hundred RMB	0.877
$E_{P}$	The compensation from the private companies to the public is assumed to be 1.1 times the benefit of opportunistic behavior. Thus, the value of $E_{P}$ is 0.836 hundred million RMB	0.836
$H_{{{\text{P}}1}}$	The environmental benefit from hardworking behavior of private companies is set at 1.2 times the value of construction cost $L_{P1}$. Thus, the value of $H_{{{\text{P}}1}}$ is estimated to be 8.2944 hundred million RMB	8.2944
$C_{C1}$	The total population of Wuhan is 12.3million. The supervision cost per person is assumed to be 10RMB, with a total value of 1.23 hundred million RMB	1.23
$E_{G}$	The value of rewards for reporting opportunistic behavior by the public is assumed to be equal to the value of opportunistic income of private companies. Thus, the initial value of $E_{G}$ is 0.76 hundred million RMB	0.76
$G_{G1}$	Under the strict regulation strategy G1, the government’s income is set to be equal to the construction investment $R_{P}$. Thus, the value of $G_{G1}$ is 8.77 hundred million RMB	8.77
$G_{G2}$	Under the loose regulation strategy G2, the government’s income is assumed to be equal to the value of discount coefficient $\omega$ times the value of $G_{G1}$. Thus, the value of $G_{G2}$ is estimated to be 7.805 hundred million RMB	7.805
$C_{G}$	The total public budget expenditure of Qingshan District in Wuhan in 2021 is 19.92 hundred million RMB. The additional Supervision cost is calculated at 10% of the total expenditure, which is 1.992 hundred million RMB	1.992
$S_{G}$	Based on the total population of Wuhan, the reputation loss for the government is assumed to be 1.23 hundred million RMB	1.23
$\omega$	We assume that the construction costs per square kilometer under strategy G1 and strategy G2 are 1.8 hundred million RMB and 1.6 hundred million RMB, respectively. Thus, the value of $\omega$ is set to be 0.89	0.89
$\eta$	The value of discount coefficient of income $\eta$ is assumed to be equal to the value of $\omega$	0.89

### Simulation analysis of evolutionary stable strategy

**Figure 13:**
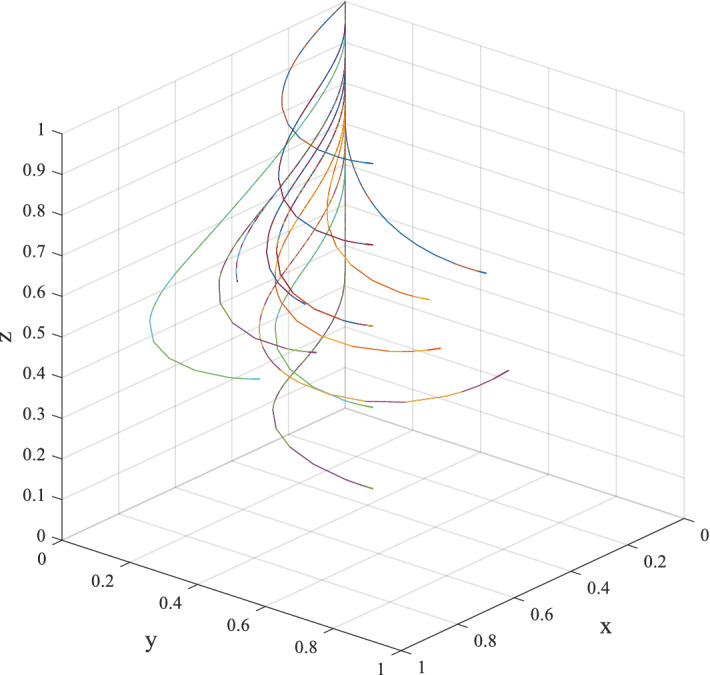
Evolutionary path diagram under various initial probabilities.

Figure 13 shows that different initial values in the game lead to different saddle points. It verifies the conclusion in Table 3 that there is no stable equilibrium point in the tripartite game system between private companies, citizens and the government. Due to the fact that the equilibrium state of the game system is not robust to the disturbance of the strategy probability of each player, so it is not feasible to induce the three players of the game to reach each expected steady state by trying different initial conditions.

### Numerical simulation analysis of cost reference points

**Figure 14:**
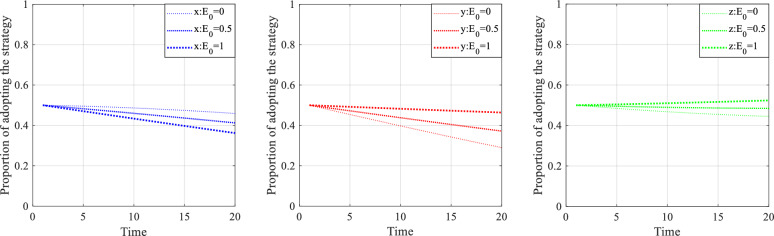
Evolution of strategies under different cost reference values $E_{0}$.

Figure 14 shows the behavior evolution of the private companies, citizens and the government under different cost reference points $E_{0}$. The lines get thicker as the value of $E_{0}$ increases from 0 to 1. As seen in Fig. 14, the probability of $x$ gradually decreases as the increases of $E_{0}$, indicating that the high value of cost reference reduces the perception of punishment and is not conducive to the effort behavior of private companies. The probabilities of $y$ and $z$ gradually increase with the increase of $E_{0}$, which implied that the low perceived value of supervision cost can effectively promote the public and the government to participate in project supervision. Therefore, psychological reference point plays an important role in the management of SC projects.

### Numerical simulation analysis of government punishments

**Figure 15:**
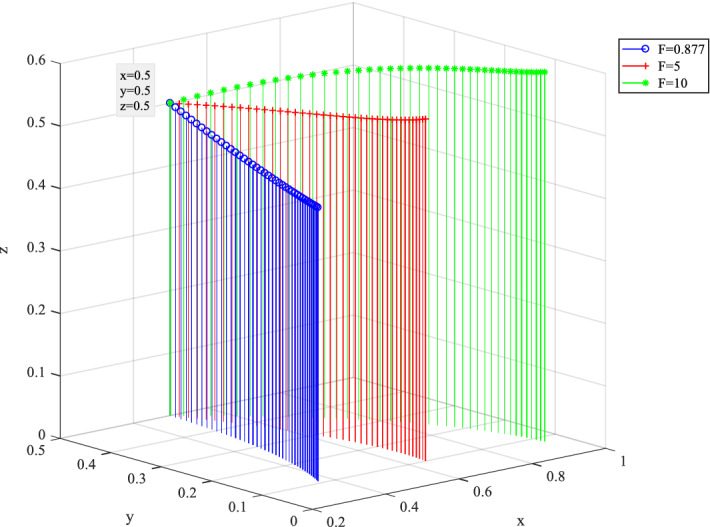
Behavior evolution of three players under different government punishments $F$.

Behavior evolution of three players under different government punishments $F$ is shown in Fig. 15. The value of $F$ ranges from 0.877 to 10. With the increase of $F$, the probabilities of the $x$ and $z$ converge to 1. It indicates that the high perceived value of punishment promotes effortful behavior and reduces the probability of opportunistic behavior by private companies. Meanwhile, it helps the government to conduct strict supervision and regulate the behavior of private companies. Therefore, the high perceived value of punishments promotes and standardizes the sound operation of SC PPP projects.

### Numerical simulation analysis of supervision cost

**Figure 16:**
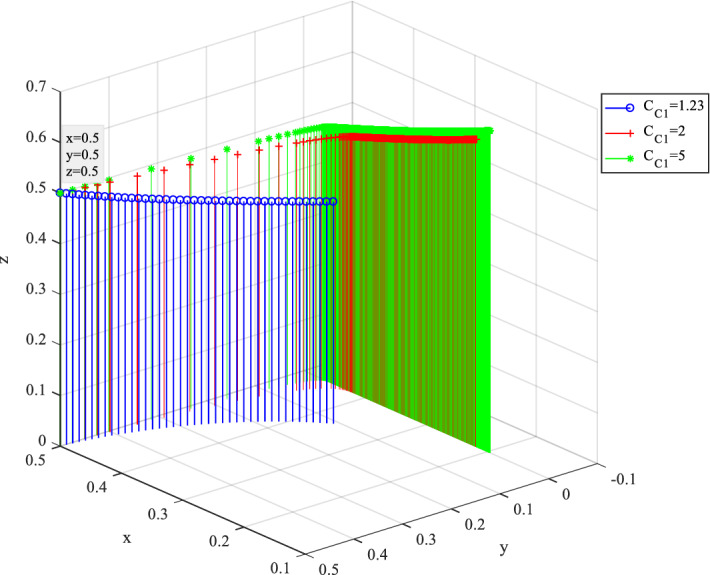
Behavior evolution of three players under different supervision cost $C_{C1}$.

Behavior evolution under different supervision cost $C_{C1}$ is shown in Fig. 16, where $C_{C1}$ is ranged from 1.23 to 5. With the increase of the supervision cost $C_{C1}$, $y$ gradually coverages to 0. It reveals that the enthusiasm of public participation in supervision is closely related to the cost of supervision. Under high perceived cost of supervision, the probability of citizens choosing strategy C2 reduces. Meanwhile, the probability of $z$ increases as the value of $C_{C1}$ increases. It implies that under the scenario of low probability of public participation in supervision, the government tends to choose strict regulation strategies to ensure the management and control of SC projects.

### Simulation analysis of the discount coefficient

**Figure 17:**
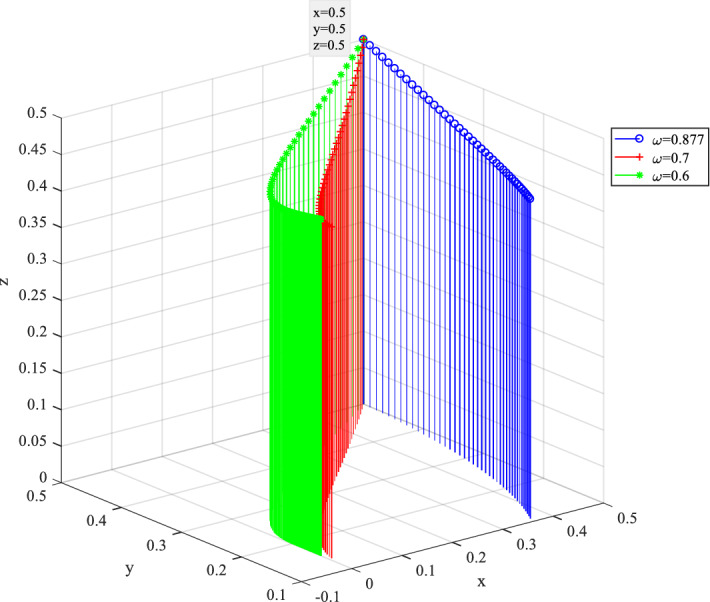
Behavior evolution of three players under different discount coefficient $\omega$.

Impact of the discount coefficient of cost $\omega$ on the behavior evolution is shown in Fig. 17, where $\omega$ ranges from 0.877 to 0.6. The decrease of $\omega$ indicates that private companies reduce the construction investment, leading to the worse consequences. $x$ rapidly converges to 0 as $\omega$ decreases from 0.877 to 0.6. The lower cost of construction due to opportunistic behavior and the resulting high benefits decrease the probability of hardworking construction for private companies. In this case, the probability of $y$ and $z$ gradually increases. Facing the opportunistic behavior of private companies, the two-tier regulation system between the government and the public is helpful for the management of opportunistic construction and cover corresponding losses in time.

### Simulation analysis of reputation loss

**Figure 18:**
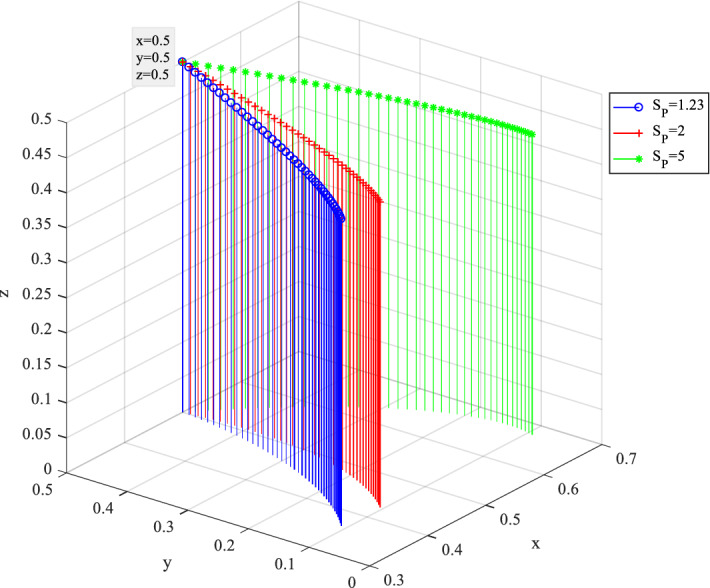
Behavior evolution of three players under different reputation loss $S_{P}$.

Behavior evolution under different reputation loss $S_{P}$ is shown in Fig. 18, where $S_{P}$ is ranged from 1.23 to 5. It showed that with the rise of reputation loss, $x$ gradually coverages to 1, thus, private companies tend to adopt the hardworking construction strategy P1. Although the perceived value of reputation loss caused by opportunistic behavior is difficult to quantify in business operations. This simulation revealed that with the increasing social impact and public resistance caused by reputation loss, private enterprises are not inclined to take opportunistic behavior in the construction of SC project.

### Results

Theoretically, when all three participants are actively involved in the construction process, it is the ideal state of the system and contributes to the development of SC. However, it can be seen that this game model cannot always achieve the evolutionary stable state of $(1,1,1)$. The influence factors that prevent this game model reaching its ideal state are explained as follows:

First, the perceived cost of behavior is high and the corresponding perceived incomes of behavior is low. There are different reference points $(E_{0} ,E_{1} )$ for cost and income accounts. McGregor and Cutcher-Gershenfeld^50^ claimed that people want high returns through low effort. Thus, there are low value of cost reference $E_{0}$ and high value of income reference $E_{1}$ in mental accounting of game participants. As a result, the psychological perception of loss fluctuation is greater than that of gain. For the income account, the perceived gains of hardworking construction and regulatory behavior are comparable to that of opportunistic construction and non-regulatory behavior. As for loss accounts, the low value of $E_{0}$ leads to a strong perception of cost, which increases its tendency to choose low-cost behavior. Therefore, private companies tend to choose opportunistic strategy P2, and citizens and the government not incline to participate in project supervision.

Second, participants are prone to have fluke mind and optimism bias when making judgments about strategic choices. People think they are less likely to encounter negative events than other people, and tend to assume that things will develop towards good results^51^. Optimistic bias reduces people’s risk perception and problems solving efforts^52^. According to Prospect Theory, the probability of high probability events is underestimated, as $\pi (p) \le p$. For private companies, the probability of public participation in supervision and the probability of strict government supervision are often underestimated due to the subjectivity of cognition, that is, $\pi (y) < y$ and $\pi (z) < z$. Thus, driven by fluke mind, private companies believe that there are loopholes in the regulation system, which leads to the opportunistic behavior. Similarly, from the perspective of the government, it tends to believe that private companies are unlikely to take opportunistic due to the compliance with policy regulations, that is, $\pi (x) < x$. Therefore, the government is likely to choose loose regulation strategy G2.

Third, loss avoidance has a significant impact on psychological accounts in the risk decision process. Reason^53^ believed that unconscious unsafe behavior stems from lack of cognitive ability, while opportunistic behavior originates from risk decision-making. Wang and Johnson^54^ further illustrated the different attitudes of risk in the face of incomes and costs. Prospect theory^31^ suggested that people are risk averse for income accounts. As for loss accounts, people have an appetite for risk. When facing income account, private companies are risk averse, with small values of $\chi$ and $\zeta$, which weakens the difference in income perception between the P1 and P2 strategies. However, in the cost account, the value of cost represents the perception of loss. In this condition, private companies are risky pursuits, with a larger value of $\tau$ and $\kappa$. The strong loss aversion psychology strengthens the perceived value of the cost. When private companies choose strategy P1 to work hard, there will be a definite cost $L_{P1}$. With the increase of the perceived loss of $L_{P1}$, private companies tend to choose the low-cost strategy P2. Similarly, citizens and the government also have risk aversion and risk pursuit psychology when confronting the strategic choice, and the risk pursuit is more obvious. It requires the certain cost of $C_{C1}$ and $C_{G}$ to participate in regulation respectively. Therefore, loss aversion decreases the probability that citizens and the government choose to participate in management.

## Discussions

The analysis and simulation showed that there is no ESS in this game model. The result indicates that in the process of SC construction, the strategic choice of participants fluctuates with the strategies of other participants. It reflects the problems existing in China under the present regulatory mode of engineering projects. When opportunistic behaviors of private companies occur frequently, the government and the public tend to pay more attention to the project construction and strengthen supervision. Then private companies would work harder to build projects during the period. However, with the benign construction atmosphere of the project, the government and the public would gradually adopt loose supervision considering the supervision cost. Accordingly, private companies are inclined to gradually appear opportunistic behaviors, which in turn aggravate the hidden quality problems of SC project construction.

Traditional measures to prevent opportunistic behavior are based on expected utility theory, which holds that the decision-making of participants is based on absolute returns. Therefore, the government focuses on strengthening government supervision, improve the high punishment mechanism. Since the influence of the psychological factors of each participant on decision-making is not considered, there is a deviation between the traditional regulatory system and reality. Both theories and practice have proved that simple punishment is not effective in preventing opportunistic behaviors. In fact, the behavior mechanism of participants is complex, which is affected by many factors such as risk attitude, loss aversion coefficient and psychological reference point. According to Prospect Theory and Mental Accounting, risk decisions depend heavily on whether the decision maker focuses on income or cost, that is, the perception of income and cost is different. The participants make decisions based on the perceived value determined by the subjective value of mental accounting and the decision weights. Thus, the main factor influencing the decision is not absolute income but the subjective perception in mental accounting. The government should change its way of thinking and focus on the perspective of perceived value to develop reasonable guidance.

Based on the above analysis and discussion, the following management recommendations for SC projects are proposed.

1. The government should gradually improve the reputation system construction of project participants and ensure the accuracy and authority of information. It can effectively increase the perceived cost of reputation loss $u_{C} \left( {S_{P} } \right)$, and produce a corresponding deterrent effect on opportunistic behavior. Furthermore, the promotion of high reputation corporate image can also help guide a positive atmosphere in the engineering field, reduce the psychological preference of private enterprises for risky behavior, and form a harmonious and upward industry trend.
2. Strengthen the exposure of SC construction details. The construction of SC projects is complicated, and the detailed information of construction cost and progress of each link has not formed a perfect unified platform for publicity. The process of public access to information is cumbersome, which invariably increases the perceived cost of regulation. Moreover, a unified platform control facilitates horizontal and vertical comparison of construction efficiency, and also serves the purpose of psychological deterrence of opportunistic behavior.
3. Improve the supervision system of public participation. The Chinese government plans to transform 80% of built-up urban areas into SC by 2030. There is a large group of private enterprises involved in construction, and the government’s supervision is limited by the cost of supervision, so it is impossible to conduct comprehensive supervision. And the potential of the public is huge. Therefore, improving the reporting mechanism not only facilitates the way for the public to protect their rights, but also reduces regulatory pressure of the government.

## Conclusions

Sponge City PPP projects are an important strategy to promote the sustainable development of urban water ecology and the construction of resilient city in China. The opportunistic behavior in project construction is an issue that all social parties are concerned about. However, existing regulatory policies of SC PPP projects ignore the great role of the public. In the exploratory research related to the urban governance, scholars emphasized the importance of the civic society in promoting the benign operation of PPP projects by constructing quadruple-helix model^55^ and penta-helix model^56^. Therefore, it is of great significance to study the evolutionary mechanism of opportunistic behavior under the supervision of the government and citizens, and contributes to the future management of SC projects. Considering the influence of risk preferences under the bounded rational of game players, this paper introduced Prospect Theory and Mental Accounting to construct the perceived value game matrix, and verified the results through numerical simulation, which can reflect the game situations of main stakeholders. The findings of this study make several contributions to the current literature.

1. This paper provides the first research on opportunistic behavior of SC PPP projects based on the perspective of micro perceived value and accordingly proposes the management recommendations to improve the governance effect of SC PPP projects, which not only provides new ideas for the construction supervision and policy-making of SC projects, but also serves as reference for governance in other engineering fields.
2. Model analysis and numerical simulation show that there is no equilibrium stable state in the game between private companies, citizens and the government. The strategy evolution of tripartite game system affects each other and is affected by perceived incomes and costs, which is a dynamic evolutionary process.
3. Under the premise of bounded rationality of the participants, the root cause of their negative attitude towards SC construction and supervision is that the perceived costs are greater than the perceived benefits obtained by adopting positive actions.
4. This paper reveals the influence of reference value on the decision-making behavior. The high reference point of cost weakens the participant’s perceived value of cost. For private companies, the low perceived value of penalty hinders their choice of hardworking construction strategy P1. Meanwhile, low perceived value of regulatory costs promotes the public and government participation in the governance of SC projects.
5. Government punishment and reputation loss can effectively curb the opportunistic behavior of private companies. Increasing the perceived cost caused by tangible punishment and intangible reputation loss is effective measure to promote the moral behavior of private companies.

Nevertheless, this paper still has some limitations. Firstly, the model constructed in this paper is a general model with influencing parameters extracted from existing empirical literature. It is difficult to consider the diversity and heterogeneity between different SC projects. This paper focuses on the analytical framework of opportunistic behavior regulation, especially the impact of the interaction between participants. Secondly, in the aspect of participants selection, only three overall participants are considered without further detailed division, while the actual urban governance is a complex system with many participants. For example, penta-helix model emphasizes the importance of the academy, civic society, and social entrepreneurs/activities groups in enhancing the democratisation in smart cities^56^. This paper may simplify the real situation, but it does not affect the logical framework of this article. SC projects, as a complex artificial system, involved in many subjects and complex social relationship network. Future work can further explore the optimal regulation and incentives measures of SC projects under the manipulation of complex psychological factors, and discuss about the different supervision effects under different specific participants.

## Supplementary Information

Supplementary Information.
