# Supplementary material for: Evolutionary game analysis of opportunistic behavior of Sponge City PPP projects: a perceived value perspective
Source: Sci Rep. 2022 May 25;12:8798. doi: 10.1038/s41598-022-12830-0 (PMC9132952; doi:10.1038/s41598-022-12830-0)
Supplement: Supplementary file 1 — Supplementary Information. [file 41598_2022_12830_MOESM1_ESM.doc]

**Appendix A. The solutions of the dynamic replication equations**

When private companies choose strategy P1 of “hardworking behavior”, the perceived utility is defined as follows：

| $\begin{matrix} V_{P1}=\pi(y)\pi(z)\left[u_{I}(R_{p})-u_{C}(L_{P1})\right] \\ +\pi(y)\pi(1-z)\left[u_{I}(R_{p})-u_{C}(L_{P1})\right] \\ +\pi(1-y)\pi(z)\left[u_{I}(R_{p})-u_{C}(L_{P1})\right] \\ +\pi(1-y)\pi(1-z)\left[u_{I}(R_{p})-u_{C}(L_{P1})\right] \end{matrix}$ | (A.1) |
| --- | --- |

Where $V_{P1}$ represents the perceived utility of private companies choosing the strategy “hardworking behavior”. When private companies choose strategy P2 of “opportunistic behavior”, the perceived utility is defined as follows：

| $\begin{matrix} V_{P2}=\pi(y)\pi(z)\left[u_{I}(R_{p})-u_{C}(\omega L_{P1}+F+E_{P})\right] \\ +\pi(y)\pi(1-z)\left[u_{I}(R_{p})-u_{C}(\omega L_{P1}+S_{P})\right] \\ +\pi(1-y)\pi(z)\left[u_{I}(R_{p})-u_{C}(\omega L_{P1})\right] \\ +\pi(1-y)\pi(1-z)\left[u_{I}(R_{p})-u_{C}(\omega L_{P1})\right] \end{matrix}$ | (A.2) |
| --- | --- |

Where $V_{P2}$ represents the perceived utility of private companies choosing the strategy “opportunistic behavior”. Thus, the expected perceived utility of private companies $(\bar{V}_{P})$ is：

| $\bar{V}_{P}=xV_{P1}+(1-x)V_{P2}$ | (A.3) |
| --- | --- |

Similarly, when citizens choose strategy C1 of “public supervision”, the perceived utility can be described as follows:

| $\begin{matrix} V_{C1}=\pi(x)\pi(z)\left[u_{I}(H_{P1})-u_{C}(C{}_{C1})\right] \\ +\pi(x)\pi(1-z)\left[u_{I}(H_{P1})-u_{C}(C{}_{C1})\right] \\ +\pi(1-x)\pi(z)\left[u_{I}(E_{P}+E_{G}+\eta H_{P1})-u_{C}(C_{C1})\right] \\ +\pi(1-x)\pi(1-z)\left[u_{I}\left(\eta H_{P1}\right)-u_{C}(C_{C1})\right] \end{matrix}$ | (A.4) |
| --- | --- |

Where $V_{C1}$ denotes the perceived utility of citizens choosing the strategy “public supervision”. When citizens choose strategy C2 of “no public supervision”, their perceived utility is defined as follows：

| $\begin{matrix} V_{C2}=\pi(x)\pi(z)\left[u_{I}(H_{P1})\right] \\ +\pi(x)\pi(1-z)\left[u_{I}(H_{P1})\right] \\ +\pi(1-x)\pi(z)\left[u_{I}(\eta H_{P1})\right] \\ +\pi(1-x)\pi(1-z)\left[u_{I}(\eta H_{P1})\right] \end{matrix}$ | (A.5) |
| --- | --- |

Where $V_{C2}$ denotes the perceived utility of citizens choosing the strategy “no public supervision”. Thus, the expected perceived utility of citizens $(\bar{V}_{C})$ is：

| $\bar{V}_{C}=yV_{C1}+\left(1-y\right)V_{C2}$ | (A.6) |
| --- | --- |

Accordingly, when governments choose strategy G1 of “strict regulation”, the perceived utility can be calculated as follows：

|  | (A.7) |
| --- | --- |

Where $V_{G1}$ denotes the perceived utility of governments choosing strategy “strict regulation”. When the government choose strategy G2, the perceived utility is defined as follows：

| $\begin{matrix} V_{G2}=\pi(x)\pi(y)\left[u_{I}(G_{G2})-u_{C}(R_{P})\right] \\ +\pi(x)\pi(1-y)\left[u_{I}(G_{G2})-u_{C}(R_{P})\right] \\ +\pi(1-x)\pi(y)\left[u_{I}(G_{G2})-u_{C}(R_{P}+S_{G}+\left(1-\omega\right)L_{P1})\right] \\ +\pi(1-x)\pi(1-y)\left[u_{I}(G_{G2})-u_{C}(R_{P}+\left(1-\omega\right)L_{P1})\right] \end{matrix}$ | (A.8) |
| --- | --- |

Where $V_{G2}$ denotes the perceived utility of governments choosing strategy “loose regulation”. Thus, the expected perceived utility of governments $(\bar{V}_{G})$ is：

| $\bar{V}_{G}=zV_{G1}+(1-z)V_{G2}$ | (A.9) |
| --- | --- |
